# Analysis of the Full-Length Pyriform Spidroin Gene Sequence

**DOI:** 10.3390/genes10060425

**Published:** 2019-06-03

**Authors:** Kangkang Wang, Rui Wen, Qiupin Jia, Xiangqin Liu, Junhua Xiao, Qing Meng

**Affiliations:** 1 Institute of Biological Sciences and Biotechnology, Donghua University, Shanghai 201620, China; kangkangwong594@gmail.com (K.W.); 1162014@mail.dhu.edu.cn (R.W.); 1132046@mail.dhu.edu.cn (Q.J.); xiaojunhua@dhu.edu.cn (J.X.); 2 Department of Biochemistry and Molecular Biology, Dalhousie University, Halifax, NS B3H 4R2, Canada; pxqliu@dal.ca

**Keywords:** spider silk, PySp1, pyriform spidroin, gene family, full-length gene

## Abstract

Spiders often produce multiple types of silk, each with unique properties suiting them to certain tasks and biological functions. Orb-weaver spiders can generate more than six types of silk fibroins, with pyriform silk used to form attachment discs, adhering silk to other surfaces and substances. The unique higher-order structuring of silk fibroins has been cited as the source of their remarkable biomechanical properties. Even so, only one full-length gene sequence of pyriform silk protein 1 (PySp1) from *Argiopeargentata* has been reported, and studies on the mechanical properties of natural pyriform silk fibers are also lacking. To better understand the PySp1 family of genes, we used long-distance PCR (LD-PCR) to determine the sequence of PySp1 in the *Araneusventricosus* species. This full-length PySp1 gene is 11,931 bp in length, encoding for 3976 amino acids residues in non-repetitive N- and C-terminal domains with a central largely repetitive region made up of sixteen remarkably homogeneous units. This was similar to the previously reported *A. argentata* PySp1 sequence, with PySp1 from *A. ventricosus* also having a long repetitive N-linker that bridges the N-terminal and repetitive regions. Predictions of secondary structure and hydrophobicity of *A. ventricosus* PySp1 showed the pyriform silk fiber’s functional properties. The amino acid compositions of PySp1 is obviously distinct from other spidroins. Our sequence makes an important contribution to understand pyriform silk protein structure and also provides a new template for recombinant pyriform silk proteins with attractive properties.

## 1. Introduction

With differing and intriguing high-performance, spider silk can be tougher and lighter than other natural fibers and synthetic fibers [1]. The advanced female orb-weaving spiders spin more than six different silk fiber types, also producing watery glue from seven different types of silk glands, with each specific type of silk having properties suited to its particular roles [2,3,4]. This intense research has long been focused on the major ampullate silk (safety thread) which is used as a safety line in case of a fall and is responsible for the outer frame and spiral radii of the orb-web, with toughness that rivals the toughest known materials [5,6,7].Other silks, such as those secreted by the minor ampullate glands, express temporary capture spiral silks which provide additional web stabilization [8,9]; Flagelliform (Flag) or capture silk is synthesized in flabelliform glands, and functions as a highly elastic fiber that can ensnare rapidly moving insects which come in contact with the web [3,10]; Acini form silk serves to wrap prey and to form the inner liner of the egg case, while tubuli form silk forms its tough outer shell [2,11,12,13,14]; compared with other glands, aggregate glands produce a silk glue that is used for coating spiral threads to better enable the core fiber to capture prey [15,16]; Pyriform silk is a two-compound material including the silk fiber and cement-like glue produced in pyriform glands simultaneously [17,18].

As a very different composite silk, the pyriform silk has its own particular function. The pyriform silk forms the so-called attachment disc which is used for bonding fibers together, ensuring the whole web framework of spider robust attached to substrates, and connecting dragline silk to surfaces for escaping rapidly [19]. However, cobweb-weaving spiders produce different attachment discs with more functions, such as prey capture, locomotion, and egg sacs [17]. The distinct web architecture produces strong adhesion to affix the web to substrate, even on a very smooth surface provided [20].

The strong adhesive of pyriform silk is the result of the unique protein sequence and micro structure. Previous studies have focused on the morphology of attachment silk and the molecular aspect [18,19,20,21,22]. Scanning electron microscopy revealed that the attachment discs are composed of fibers which are small in diameter and are embedded in a gelatinous substance that has very strong adhesive properties upon drying [21]. Different from the other silk forms which can be spun as wet glues or dry fibers, the architecture of pyriform silk reveals it is a combination of both such materials. Furthermore, the glue and fibers originate from the same glands (pyriform glands) [18,22]. Spider silk fibroins are very large, highly repetitive, and homogenized by type within a species. In the case of the major ampullate and minor ampullatespidroins (MaSp and MiSp, respectively), the repetitive regions contain short, simple repeat units. However, the aciniform and tubuliformspidroin (AcSp and TuSp, respectively) genes encode longer, much more complicated repeats [12,14,23,24,25]. The reported pyriform spidroin (PySp) sequences have similar primary structure with AcSp and TuSp families. For those spidroins (AcSp, TuSp, and PySp), many long repetitive sequences (>200 amino acids) are surrounded by short amino (N)- and carboxyl (C)- terminal regions, which are not repetitive. The number of repetitive units dominate the length of protein sequence. Nonetheless, Pyriform silk proteins have been discovered fairly recently, and prior to the present study, only one complete PySp1 gene sequence of *Argiopeargentata* was identified in 2017 [26].

As anchoring silk, it has unique molecular mechanisms to accomplish these tasks. Studies have reported that these proteins have alternating proline- and glutamine-rich motifs, each of which are 6–8 amino acids long, as has been shown for the pyriform silk of three species: *Argiope trifasciata*, *Nephila clavipes*, and *Nephilengyscruentata* [27]. As a rather unexplored silk cement, the pyriform silk protein composition remains largely unclear. The basic composition of the PySp1 encoding protein is worth more exploring. More PySp1 gene sequences are still necessary. To better understand the properties of spider silk proteins, we herein derive the full-length *Araneusventricosus* PySp1 sequence via long distance PCR (LD-PCR).

## 2. Materials and Methods

### 2.1. PySp1 Degenerate PCR

Members of the *A. ventricosus* species were located and collected in Shanghai, China, and were rapidly frozen using liquid nitrogen, followed by storage at −80°C. The cephalothoraxes of ten specimens were then used to isolate high molecular weight genomic DNA (HMW-gDNA) using the Rapid Animal Genomic DNA Isolation Kit (Sangon, Shanghai, China) and treated with RNase A.

According to the N- and C-terminal amino acid sequences of *A. argentata*, and using PySp1 and partial repetitive region sequences of *A. ventricosus* PySp1 that we obtained previously, we constructed 2 degenerate primers as well as 2 gene-specific primers to be used in a degenerate PCR reaction for these regions (Table 1). A sixamino acids region (KSWVQD) in the N-terminal domain was chosen for the forward degenerate primer, while the repetitive region was targeted by the reverse gene-specific primer to amplify the partial N-terminal region (Table 1). Another degenerate primer (GGQVNY) was designed in the C-terminal region for degenerate reversed primer, together with the forward gene-specific primer, used for partial C-terminal amplification (Table 1). In order to obtain the target sequences, agarose gel electrophoresis was used to isolate fragments of multiple different sizes from degenerate PCR. These fragments were gel-extracted and sequenced, and then aligned with other published PySp1 sequences to identify which one is target sequence. Sequencing of these initial DNA products was used to generate 2 pairs of gene-specific primers for the anchor PCR, as follows: 5′-TTT TCA ATA GCT GCC GCC TGC-3′ and 5′-CTC GAA CAC CTG TTG TTG CTT C-3′, as well as the anchor primer 5′-ACT CCT GTG GAA CCA TCG GAC GGG GGG-3′ were utilized in order to amplify the 5′ end sequence of the N-terminal region. The gene-specific primers 5′-GCA ATC TTC CGT TGC TCA ATC TC-3′ and 5′-TTC AAG CGG AGC TTC ATC AGG-3′ were utilized to amplify the 3′ end sequence of the C-terminal region, in accordance with the above anchor primer. The method for obtaining target sequences was as described above.

### 2.2. Long-Distance PCR for Full-length PySp1 Gene

Two gene-specific primers were constructed as a means of amplifying the full-length PySp1 gene. A forward primer 5′-AGC GAT GTC TTG GAC CCT GGG GCT TC-3′ was designed in N-terminal region, while a reverse primer5′-CTA TCC AAG TGC TGC AAG TAC G-3′ was designed in the C-terminal region. Because of the large size of PySp1, we used Phusion high-fidelity DNA polymerase (NEB Beijing, China) to guarantee precise LD-PCR amplification. The reaction was conducted using the following conditions: 98 °C for 1 min, 30 amplification cycles (98 °C for 5 s; 56 °C for 20 s; 72 °C for 10 min), and 72 °C for 2 min. Following the amplification of the PySp1 gene, the amplified DNA products were gel-extracted based on provided directions (Sangon, Shanghai, China) and ligated with pEASY-blunt zero cloning vector (TRANSGEN Beijing, China), followed by *Escherichia coli* transformation. PySp1 vector insertion validation was conducted based on PCR and restriction enzyme digestion in concert with agarose gel electrophoresis. Those plasmids containing a complete PySp1 gene were then sequenced by the Beijing NoVogene Bioinformatics Technology Company.

### 2.3. Sequencing and Assembly

Agarose gel electrophoresis was used to isolate DNA from plasmids, which then underwent Qubit quantification. The plasmid DNA was then sequenced via an Illumina HeSeq 4000-PE150 machine using massive parallel sequencing. Paired-end A-tailed adaptors were used for the library construction at the Beijing Novogene Bioinformatics Technology Co. Ltd., with PCR amplification of a 500 bp insert and a mate-pair library with an insert size of 5 kb.

For quality control, we removed all adaptor sequences, reads containing poly-N and low quality (with quality score to be less than 5) from the dataset using Fastp [28], and then used SOAP de novo to assemble the remaining clean reads into scaffolds, after which they were used for gap-closing.

### 2.4. Sequence Analysis

SignalP v. 4.1 was used for predicting PySp1 N-terminal signal cleavage peptide [29]. This sequence was compared to other previously reported silk protein sequences, using Geneiousv.7.1.7 for sequence alignments. For phylogenetic analyses, the N- and C-terminal domains of available silk proteins were selected if available within the NCBI database (All the accession numbers given in Supplementary Table S1) and analyzed with MEGA 6 [30]. For geneious alignment, we used the Protein Weight Matrix (Gonnet), and set the gap open penalty and gap extension penalty to 10 and 0.2, respectively. The conserved spidorinN- and C-terminal regions from the complete *A. ventricosus* PySp1 were aligned to 20 published spidroins, which also have both N- and C-terminal regions using ClustalW [31]. The N- and C-terminal regions were aligned with default setting, and the alignments were adjusted by eye, and were then concatenated for phylogenetic analysis (Figure S1). Maximum likelihood (ML) searches for best trees and bootstrap were conducted for 1,000 replicates with the Jones–Taylor–Thornton (JTT) model of amino acid substitutions. The Kyte–Doolittle method was used to approximate hydrophilicity using Expasy tools (www.expasy.org) [32], while PSIPRED v3.3 was used for secondary structure predictions [33]. DNAman and DNAssist were used to assess codon usage and amino acid composition [34].

## 3. Results

### 3.1. PySp1 Primary Structure

**Figure 1:**
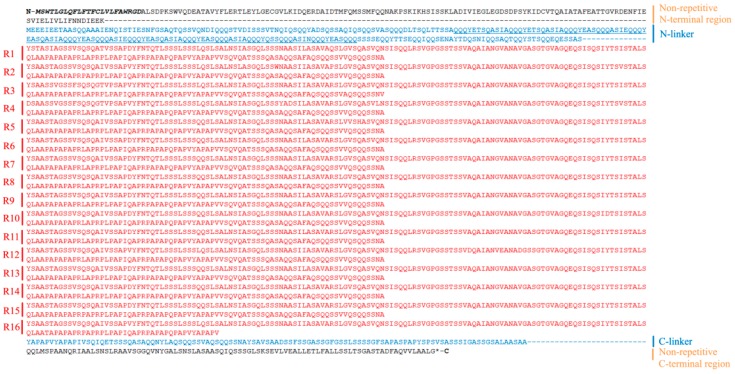
Complete *A. ventricosus* pyriform spidroin 1 (PySp1) sequence. The starting position is marked by amethioine (M), with an asterisk in the stop position. The sequence contains the non-repetitive N- and C-terminal regions, as well as two linker regions and the highly repetitive central region. Bold italics mark the signal peptide region. The underlined region is the N-linker. Gaps (-) are used for repeat unit alignment. Black is used to denote the non-repetitive N- and C-terminal, while linker regions are blue.

We sequenced and assembled one positive clone containing 11,935 base pairs (bp) from the genomic *Araneusventricosus* DNA (accession: MH376748), which contained an 11,931 bp long open reading frame (ORF) encoding a predicted 3,976 aa PySp1. No introns were detected. *A. ventricosus* PySp1 has five regions: a non-repetitive N-terminal region (150 aa), a long N-terminal linker region (256 aa), a repetitive central region that dominates ~85% of the protein (3372 aa), a short C-terminal linker region (109 aa), and a non-repetitive C-terminal region (89 aa) (Figure 1). 

### 3.2. N- and C-Terminal Regions

As silk proteins need to undergo trafficking through the endoplasmic reticulum and into secretory pathways, they contain key secretory signaling elements [10]. We thus used signalP v.4.1 to assess the N-terminal region of this PySp1 protein in order to predict the presence of a signal peptide. Our analysis predicted the presence of a signal peptide cleavage site to be located between amino acids 24 and 25 (probability score: 0.845) (Figure 1).

**Figure 2:**
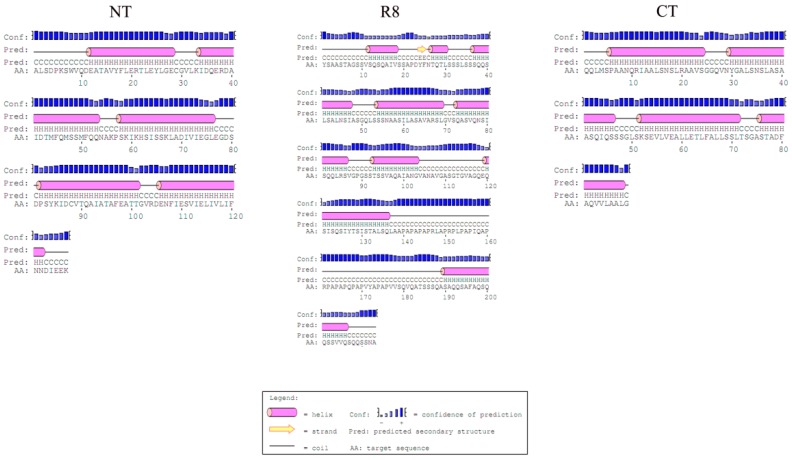
N- and C-terminal domain and repeat unit secondary structures.

The predicted N-terminal domain sequence secondary structure contains 5 α-helices in regions similar to previously reported structures [24,25] (Figure 2). The C-terminal domain, in contrast, contained just 4 α-helices (Figure 2), similar toAcSp. We further detected two N-terminal cysteines in *A. ventricosus* PySp1. The first cysteines located between helix 1 and 2, and the other one located in helix 4 (Figure 2).

**Figure 3:**
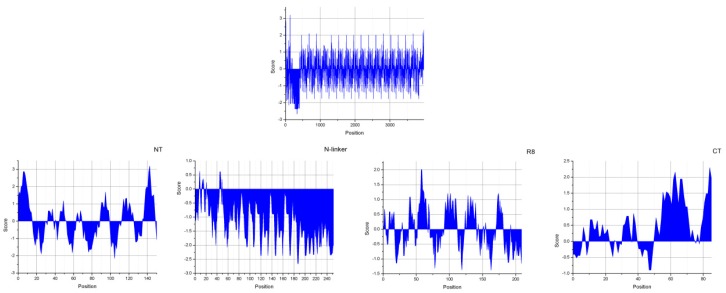
Kyte and Doolittle hydropathicity plots of *A. ventricosus* PySp1. Positive scores indicate hydrophobicity. NT: N-terminal domain, R8: repeat # 8, CT: C-terminal domain.

We further predicted *A. ventricosus* PySp1hydrophilicity, revealing an alternating profile for the entire length of this protein (Figure 3). The C-terminal domain was more hydrophobic than was the N-terminal domain when averaged across all residues (0.482 vs.0.026, respectively). The N-terminal domain did, however, have a higher hydrophobicity amplitude than did the C-terminal domain (N-terminal domain max = 3.2, C-terminal domain max = 2.3) (Figure 3).

**Figure 4:**
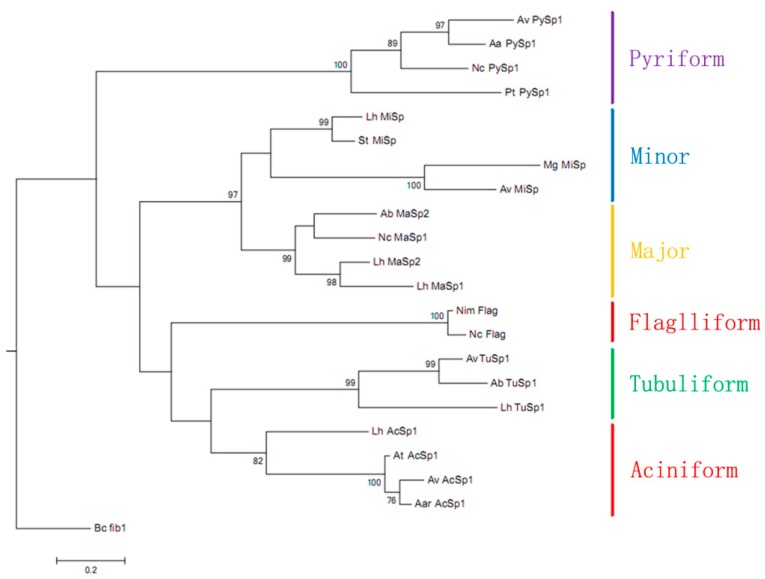
Maximum likelihood tree of N- and C-terminal domain from 21 published spidroins and *A. ventricosus* PySp1 from this study. Silk types are indicated by colored bars. Scale bar is substitution per site. Bootstrap values greater than 70% are shown. See Supplementary Table S1 for a full list of abbreviations and accession numbers.

**Figure 5:**
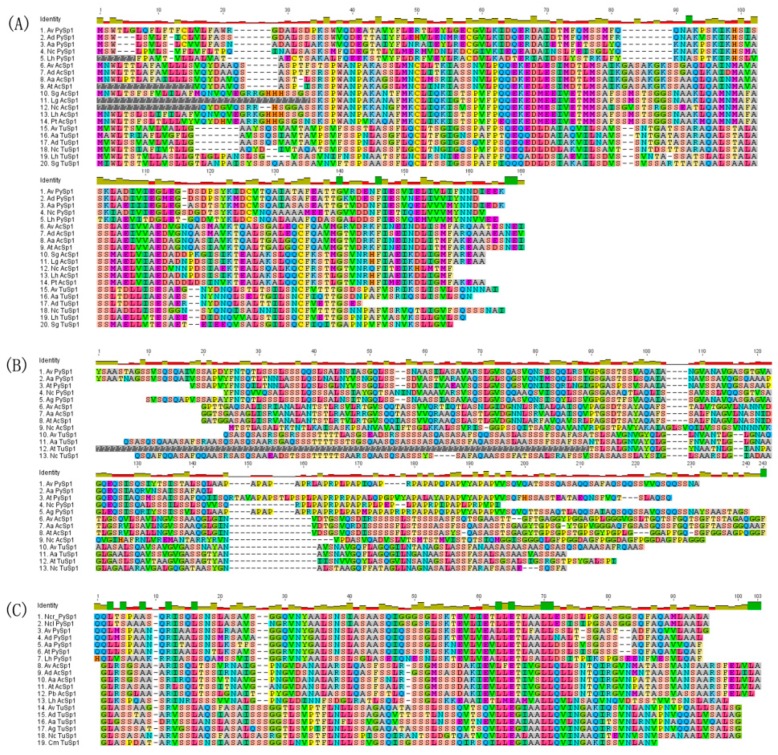
Alignment of the repeat units, N- and C-terminal domains amino acid sequences of three types of spidroins (PySp1, AcSp1, and TuSp1). (**A**,**C**) Alignment of N-terminal domain amino acid sequences (**A**) and C-terminal domain amino acid sequences (**C**). (**B**) Alignment of repeat units’ amino acid sequences. Gaps are represented by dashes. Missing data coded as “?”. The amino acid residues are represented by different colors. Species abbreviations and accession numbers in Supplementary Table S1.

To determine the phylogenetic placement of *Araneusventricosus* PySp1 within the family of silk genes, we constructed a maximum likelihood (ML) tree of concatenated N- and C-terminal amino acid sequences (Figure 4). This result shows that pyriform spidroinoriginates from a single spidroin gene family clade sister to a large grouping of AcSp, TuSp, MiSp, MaSp, and Flag sequences (Figure 4). To analyze the conservation of N- and C-terminal domains for *A. ventricosus* PySp1, we aligned these domains sequences among three types of spidroins (PySp1, AcSp1, and TuSp1) which including long and complex repeats. Multiple sequence alignment of the amino acid sequence of *A. ventricosus* PySp1 with available spider pyriform silk sequences is shown in Figure 5. In the case of the N- and C-terminal regions, *A. ventricosus* showed the closest amino acid identity with the pyriform silk protein of the spider *Araneusdiadematus* (79% and 89% identity, respectively) (Figure 5A,C). However, the alignment of repetitive sequences showed the amino acid sequences from *A. ventricosus* and *A. gemmoides* were much more closely related (84% identity) than other spiders (Figure 5B).

### 3.3. Distinctive N-linker Region

The primary molecular structure of *A. ventricosus* PySp1 exhibited two linkers (a long N-linker and a short C-linker) locate between non-repetitive terminal regions and repetitive region (Figure 1). As the same as the N-linker of *A. argentata* PySp1, the N-linker for *A. ventricosus* PySp1 contains a short 114 aarepetitive region which consists of two types of repeats (QQQYEXSQASIA and QQQYXXSQQQASIX). Glutamine (41.2%), Alanine (16.7%), and Serine (15.8%) are the most common amino acids within the repetitive N-linker. The hydrophilicity of N-linker was analyzed as above, revealing that it displays strong hydrophilicity (average = −1.202, min = −2.656) (Figure 3).

### 3.4. Core Repetitive Region

**Figure 6:**
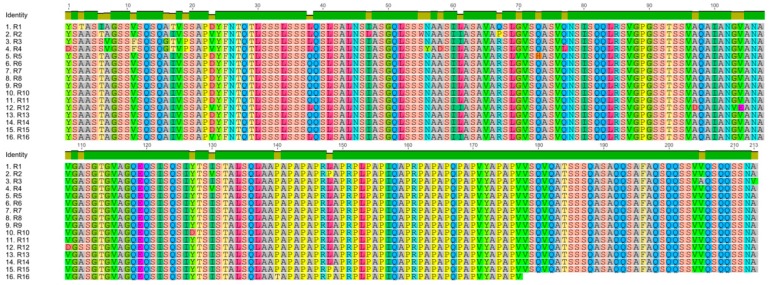
Alignment of the 16 repeat units (R1 through R16) of *Araneusventricosus* PySp1.

The repetitive portion of *A. ventricosus* PySp1is composed of 15 complete repeats (213 aa each), after which there is a partial repeat (177 aa) leading into the C-terminal linker region (Figure 1). One sequence in these repetitive motifs was particularly noteworthy and has also been identified in other pyriform fibroins (Figure 5B) [27]. The PXPXP motif is made of proline alternating with primarily alanine and arginine. However, the number of the QQ containing motif (QQxxxx), which has been identified in previouswork, is less than in other species (Figure 5B) [21].Pairwise comparisons of the repetitive units of *A. ventricosus* PySp1 revealed them to be highly conserved in terms of their amino acid and DNA sequences, as has been seen in other silk fibroins including TuSp1 and AcSp1 [12,14,23]. Many repeats have a 100% amino acid sequence identity (Figure 6). Although the repeat unit is 213 aa long, just 30 sites herein were variable in the aligned 16 repeats, with variations primarily located in the 5′ region of this terminal repeat (Figure 6). By comparing this PySp1 sequence with those of *A. argentata*, *A. gemmoides*, *N. clavipes*, and *A. ventricosus*, we found there to be a high conservation of repetitive units both within and between PySp1 orthologs (Figure 5B) (Table S2).

**Figure 7:**
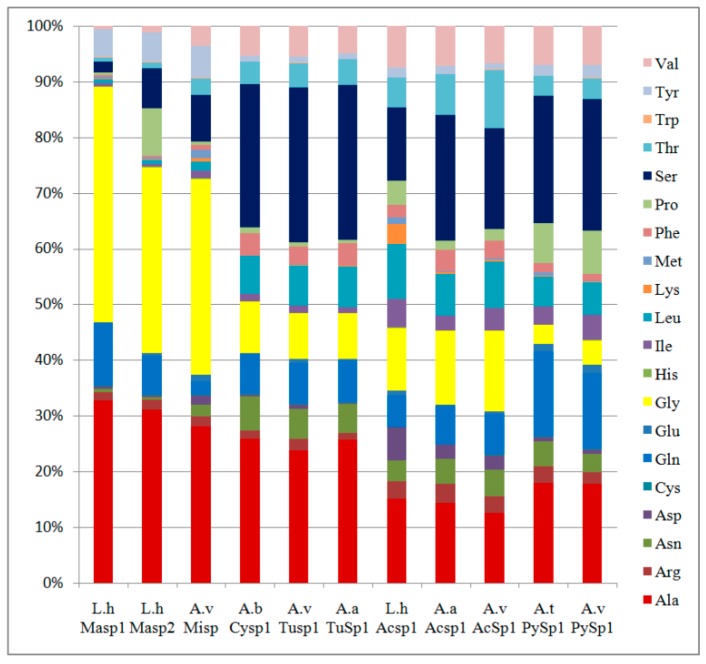
Amino acid compositions of complete L.h MaSp1, L.h MaSp2, A.vMiSp, A.b CySp1, A.v TuSp1, A.a TuSp1, L.h AcSp1, A.a AcSp1, A.v AcSp1, A.t PySp1, A.v PySp1.

Silk fibroin sequences are known to be heavily AT-biased for the third base in codons in the MaSp and MiSpsequences [23,25]. This predicted protein is expected to have a weight of ~400 kDa, and to be primarily composed of serine (23.6%), alanine (17.8%), and glutamine (13.6%) (Figure 7). This led us to assess codon usage for serine, alanine, and glycine, as well as total base composition for the coding sequences of *A. ventricosus* PySp1 (Figure S2). The result showed preferential use of A and T in alanine, glycine, and isoleucine codons. There were 626 alanines in the PySp1 sequence, and GCA codon accounted for 50% of the alanines. Of 160 glycines in the PySp1 sequence, only two were GGC. 99%of these sequenceswere encoded by GGA, GGG, and GGT. There were 143 isoleucines in the PySp1 sequence, none wereencoded by ATC. Serine codons were similarly A and T enriched in MaSp, MiSp, and Flag, whereas there was only a slight enrichment in *A. ventricosus* PySp1. Interestingly, A/T and C/G content was similar when analyzing the whole protein coding sequence, in spite of preferential A/T usage in the wobble positions.

The hydropathy profile for repeat unit of *A. ventricosus* PySp1 predicted slight hydrophilicity for each repeat unit (average = −0.015) and lower amplitude hydrophobicity than in N- and C-terminal domains (Figure 3). The predicted secondary structure of the repeat units shows that nearly half of the structures predicted are α-helices. However, the PXPXP motifs are not located in any helices (Figure 2).

Because of their extensive repetitive sequences and large sizes, and because of the 3′ bias of mRNA-based cloning techniques, few spidroin gene sequences published to data are in full length. Pyriform spidroin genes have been sequenced with more success, but to date only one annotated full-length gene sequence from *Argiopeargentata* has been made available [26]. In this study, we report the full-length *Araneusventricosus* pyriform silk DNA sequence, encoding a protein that constructs the attachment discs produced by this orb-weaving spider. We found that this gene lacks introns, and thus PySp1 possesses only one enormous exon containing 11,931 bp of coding sequence. The primary structure for *A. ventricosus* PySp1 shows that it has a highly repetitive central region between non-repetitive N- and C-terminal domains with a novel N-linker and a short C-linker (Figure 1).

As previously reported spider silk proteins for other species, *A. ventricosus* PySp1 has a high glutamine content, at 13.6% of the amino acid composition. Other such proteins, including minor ampullate and aciniform spidroins, serine, alanine, and glycine, are often the three most abundant amino acids, whereas glutamine is relatively rare (Figure 7). For example, *A. ventricosus* MiSp is predicted to be 35% glycine and just 2% glutamine. By contrast, *A. ventricosus* PySp1 is predicted to have 13.6% glutamine as well as just 4.5% glycine (Figure 7). In general, the amino acid biases of AcSp1, TuSp1, and PySp1 are slighter than MaSp and MiSp. Although not as strong as what can be seen in major and minor ampullate spidroins, biased codon usage is also observed in *A. ventricosus* PySp1. The biased codon usage in PySp1 may be due to a specific mRNA secondary structure that increases mRNA stability, and controls the silk gene transcripts as well asthe gland-specific tRNA pool. In the case of silkworm *Bombyx mori*, the biased codon usage was determined by mRNA or chromatin structure rather than tRNA population [35]. Like the *B. mori* silk gland, the major ampullate gland of *N. clavipes* develops an isoaccepting tRNA that forms with gland specificity [36]. The slight hydrophilicity of repetitive regions can be explained by the hydrophilic amino acid (Gln and Ser) region. Glutamine is thought to promote protein aggregation so that these pyriform spidroinsare able to undergo the necessary self-assembly into fibers after extrusion [21]. Therefore, it seems that glutamine-rich and more hydrophilic regions in repeat contribute to spidroin self-assembly.

Short linkers (<100 aa) are common in other spidroin [23,24]. However, the N-linkers in *A. argentata* and *A. ventricosus* PySp1 are longer in length inover 200 amino acids, with 498 aa and 256 aa, respectively (Figure 1) [26]. Moreover, the two N-linkers both contain a short repetitive region. Although the N-linker of *A. ventricosus* PySp1 contains fewer amino acids than does that of *A. argentata*, the repetitive region in N-linker from *A. ventricosus* PySp1 has similar length to *A. argentata* (114 aa vs 112 aa). The unique N-linker in PySp1 contains a glutamine-rich region that is significantly hydrophilic than other regions (Figure 3). Therefore, the function of N-linker is worth exploring. Given the previous studies [21], we hypotheses that the N-linker could regulate protein to form silk fibers or glue through affect rate of self-assembly.

The repetitive segments that compose much of the spidroin proteins have been found to be linked with the unique molecular properties of spider silk [5,8,37]. While these repetitive regions are short in some spidroins, they are longer and more complex in others [12,21,23,26]. In AcSp1 and TuSp1, as an example, the structure of these repeats is long and highly complex, being similar at the amino acid and DNA coding levels [23,38,39]. Still other spidroins including MaSp and MiSp are made up of amino acid motifs in recurring patterns called “ensemble repeats” [40]. The *A. trifasciata* and *N. clavipes*, and *A. ventricosus* pyriform spidroin repeat regions are also similar to AcSp1 and TuSp1 repeats with regard to their complexity and length (Figure 1 and Figure 6) [24,27,38,39].

The mechanical diversity of spider silk fibers islinked to the molecular structures of spider silk proteins. So far, the mechanical properties of 5 types of fibrous silks have been reported [3]. However, the mechanical performance of natural pyriform silk fibers is unknown because of its tiny structure and thee difficulty in collecting it. Our complete PySp1 sequence provides a new template for studies on mechanical properties of silk fiber through recombinant DNA technologies. Scientists have investigated the possibility of new materials by producing recombinant spider silk to specific mechanical features [41,42,43]. In addition, recently, a massive spider silk production system in *Bombyx mori* has been reported [43]. The researchers successfully replaced the silkworm fibroin heavy chain gene (*FibH*) with MaSp1 gene (1.6 kb) fused with partial *FibH* (1.1 kb) and produced 35.2% MaSp1 protein amounts in transformed cocoon shells [43]. However, these studies are all focused on the MaSp1gene. Like the MaSp gene, a desired application of PySp1 could be exploited for next generation materials. Future research could construct mini PySp1 recombinant silk protein to investigate the properties of pyriform silk fibers and prepare them for new materials.

## Figures and Tables

**Table 1:** Primers used for degenerate PCR.

	Primer Sequence (5′–3′)
**Degenerate forward primer in N-terminal region**	AARTCNTGGGTNCAGGAC
**Gene-specific reversed primer**	ACTCGCTATCGAATTGAGTGCAC
**Gene-specific forward primer**	ATCAGGAACAGGAGTTGCAGG
**Degenerate reversed primer in C-terminal region**	RTARTTNACYTGTCCTCC

## Supplementary Materials

The following are available online at https://www.mdpi.com/2073-4425/10/6/425/s1. Figure S1: Alignment of concatenated N- and C-terminal regions from sequences with both terminal regions on NCBI. N-terminal regions end at amino acid position 170 (blue line). Missing data coded as "?". 120 amino acids long per line. Species abbreviations and accession numbers in Supplementary Table S1; Figure S2: The codon usage of PySp1 gene; Table S1: Abbreviations and accession numbers used in Figure 4 and Figure 5. All accession numbers from NCBI; Table S2: The percent identity of PySp1 repetitive units among five spider species in Figure 5B.
