# Supplementary material for: Analysis of the Full-Length Pyriform Spidroin Gene Sequence
Source: Genes (Basel). 2019 Jun 3;10(6):425. doi: 10.3390/genes10060425 (PMC6627382; doi:10.3390/genes10060425)
Supplement: Supplementary file 1 [file genes-10-00425-s001.pdf]

Supplementary Table S1. Abbreviations and accession numbers used in Figure 4 and Figure 5. All accession numbers from NCBI.

Abbreviation	Full name	N-term accession	C-term accession	Repeat unit
Aa AcSp1 or Aar AcSp1	*Argiope argentata* aciniform spidroin 1	AHK09813.1		
Av AcSp1	*Araneus ventricosus* aciniform spidroin 1	AUH99620.1		
At AcSp1	*Argiope trifasciata* aciniform spidroin 1	AHK09776.1	AAR83925.1	ADN39425.1
Ad AcSp1	*Araneus diadematus* aciniform spidroin 1	AWK58687.1	AWK58698.1	
Sg AcSp1	*Steatoda grossa* aciniform spidroin 1	AWK58693.1		
Pt AcSp1	*Parasteatoda tepidariorum* aciniform spidroin 1	AWK58692.1		
Pb AcSp1	*Parawixia bistriata* aciniform spidroin 1		ADG57593.1	
Lh AcSp1	*Latrodecus hesperus* aciniform spidroin 1	AFX83557.1		
Lh TuSp1	*Latrodecus hesperus* tubuliform spidroin 1	ABD24296.1	AAY28931.1	
Ab TuSp1	*Argiope bruennichi* tubuliform spidroin 1	BAE86855.1		
Av TuSp1	*Araneus ventricosus* tubuliform spidroin	ASO67373.1		

	1			
Cm TuSp1	*Cyrtophora moluccensis* tubuliform spidroin 1		AAY28944.1	
Ad TuSp1	*Araneus diadematus* tubuliform spidroin 1	AWK58641.1	AWK58742.1	
Nc TuSp1	*Nephila clavipes* tubuliform spidroin 1	PRD35275.1		
Nc Flag	*Nephila clavipes* flagelliform spidroin	AAC38846.1	AAC38847.1	
Ag TuSp1	*Araneus gemmoide* tubuliform spidroin 1		AAX45294.1	
Nim Flag	*Nephila inaurata madagascariensis* flagelliform spidroin	AAF36091.1	AAF36092.1	
Av MiSp	*Araneus ventricosus* minor ampullate spidroin	AFV31615.1		
Mg MiSp	*Metepeira grandiosa* minor ampullate spidroin	ADM14328.1	ADM14320.1	
St MiSp	*Steatoda grossa* minor ampullate spidroin	ARA91200.1	ARA91183.1	
LhMiSp	*Latrodecus hesperus* minor	ARA91152.1		

	ampullate spidroin			
Lh MaSp1	*Latrodecus hesperus* major ampullate spidroin 1	ABR68856.1		
Lh MaSp2	*Latrodecus hesperus* major ampullate spidroin 2	ABR68855.1		
Nc MaSp1	*Nephila clavipes* major ampullate spidroin 1	ACF19412.1	AAT75312.1	
Ab MaSp2	*Argiope bruennichi* major ampullate spidroin 2	AFN54363.1		
Pt PySp1	*Parasteatoda tepidariorum* pyriform spidroin 1	XP_015910086.1	XP_015910080.1	
Nc PySp1 or Ncl PySp1	*Nephila clavipes* pyriform spidroin 1	AWK58660.1	ADN39427.1	
Aa PySp1	*Argiope argentata* pyriform spidroin 1	AQR58363.1		
Av PySp1	*Araneus ventricosus* pyriform spidroin 1	MH376748		
Sg TuSp1	*Steatoda grossa* tubuliform spidroin 1	AWK58693.1		
Ag PySp1	*Araneus gemmoides* piriform spidroin 1			AEP25627.1
Lh PySp1	*Latrodecus hesperus* pyriform spidroin 1	AWK58659.1	ACV41934.1	

Ncr PySp1	*Nephila cruentata* pyriform spidroin 1		ADK56477.1	
Bc fib1	*Bothriocyrtum californicum* fibroin 1	HM752562	EU117162	

Supplementary Table S2. The percent identity of PySp1 repetitive units among five spider species in Figure 5B.

Species	Percent of identity
Araneus ventricusos and *Argiope argentata*	64%
Araneus ventricosus and *Araneus gemmoides*	85%
Araneus ventricosus and *Nephila clavipes*	50%
Araneus ventricosus and *Argiope trifasciata*	53%
Argiope argentata and *Nephila clavipes*	55%
Argiope argentata and *Araneus gemmoides*	64%
Argiope argentata and *Argiope trifasciata*	80%
Nephila clavipes and *Araneus gemmoides*	55%
Nephila clavipes and *Argiope trifasciata*	47%
Araneus gemmoides and *Argiope trifasciata*	57%

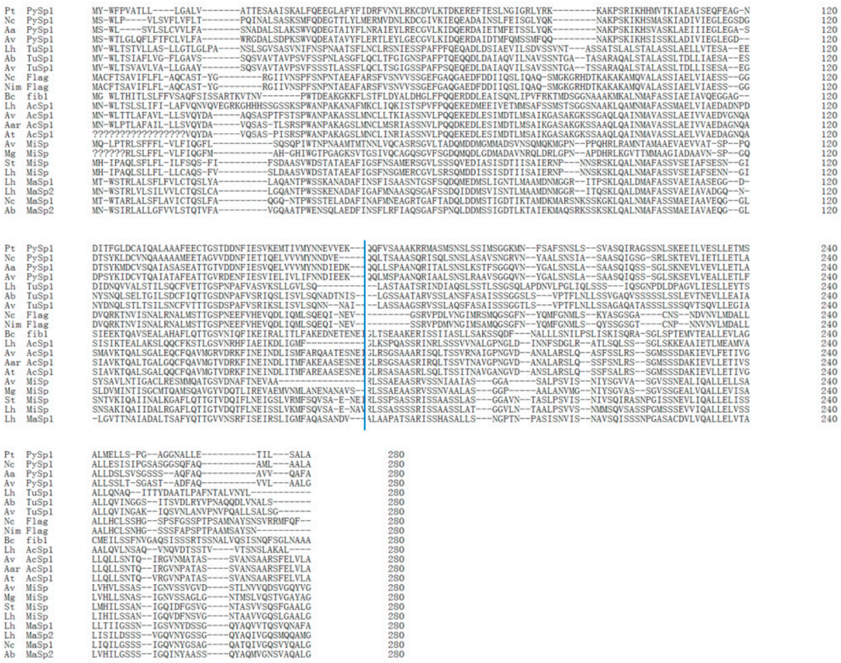

Figure S1. Alignment of concatenated N- and C-terminal regions from sequences with both terminal regions on NCBI. N-terminal regions end at amino acid position 170 (blue line). Missing data coded as "?". 120 amino acids long per line. Species abbreviations and accession numbers in Supplementary Table S1.

```
Codon usage:
gca  Ala(A)  312  # cag  Gln(Q)   92  # uug  Leu(L)   16  # uaa  Ter(.)    0
gcc  Ala(A)  107  # ---  Gln(Q)  430  # ---  Leu(L)  193  # uag  Ter(.)    0
gcg  Ala(A)   65  # gaa  Glu(E)   16  # aaa  Lys(K)    0  # uga  Ter(.)    0
gcu  Ala(A)  142  # gag  Glu(E)    1  # aag  Lys(K)    0  # ---  Ter(.)    0
---  Ala(A)  626  # ---  Glu(E)   17  # ---  Lys(K)    0  # aca  Thr(T)    6
aga  Arg(R)   61  # gga  Gly(G)  110  # aug  Met(M)    0  # acc  Thr(T)    0
agg  Arg(R)    0  # ggc  Gly(G)    2  # ---  Met(M)    0  # acg  Thr(T)   33
cga  Arg(R)    3  # ggg  Gly(G)   18  # uuc  Phe(F)   15  # acu  Thr(T)   90
cgc  Arg(R)    0  # ggu  Gly(G)   30  # uuu  Phe(F)   18  # ---  Thr(T)  129
cgg  Arg(R)   14  # ---  Gly(G)  160  # ---  Phe(F)   33  # ugg  Trp(W)    1
cgu  Arg(R)    0  # cac  His(H)    1  # cca  Pro(P)  149  # ---  Trp(W)    1
---  Arg(R)   78  # cau  His(H)    0  # ccc  Pro(P)   33  # uac  Tyr(Y)   16
aac  Asn(N)   47  # ---  His(H)    1  # ccg  Pro(P)   45  # uau  Tyr(Y)   47
aau  Asn(N)   63  # aua  Ile(I)   67  # ccu  Pro(P)   66  # ---  Tyr(Y)   63
---  Asn(N)  110  # auc  Ile(I)    0  # ---  Pro(P)  293  # gua  Val(V)   21
gac  Asp(D)    0  # auu  Ile(I)   76  # agc  Ser(S)   99  # guc  Val(V)   47
gau  Asp(D)   16  # ---  Ile(I)  143  # agu  Ser(S)  123  # gug  Val(V)   60
---  Asp(D)   16  # cua  Leu(L)   18  # uca  Ser(S)  172  # guu  Val(V)  131
ugc  Cys(C)    0  # cuc  Leu(L)   32  # ucc  Ser(S)  124  # ---  Val(V)  259
ugu  Cys(C)    0  # cug  Leu(L)   54  # ucg  Ser(S)   78  # nnn  ???(X)    0
---  Cys(C)    0  # cuu  Leu(L)   44  # ucu  Ser(S)  224  # TOTAL        3372
caa  Gln(Q)  338  # uua  Leu(L)   29  # ---  Ser(S)  820  #
```

Figure S2. The codon usage of PySp1 gene.
